# Supplementary material for: Improving Wolbachia-based control programs in urban settings: Insights from spatial modeling
Source: PLoS Negl Trop Dis. 2025 Dec 12;19(12):e0013787. doi: 10.1371/journal.pntd.0013787 (PMC12714238; doi:10.1371/journal.pntd.0013787)
Supplement: S1 Text — (PDF) [file pntd.0013787.s001.pdf]

S1 Text Supporting Information

A Cylindrically Symmetric System for 2-D Releases

To account for the radial expansion effect at which the *Wolbachia* wave propagates, we simplify the model in 3a–3b by assuming cylindrical symmetry of the solution. Therefore, the spatial diffusion is defined as $\Delta_r = \frac{\partial^2}{\partial r^2} + \frac{1}{r}\frac{\partial}{\partial r}$. For this purpose, let us consider our state variables as $u = u(r,t)$ and $w = w(r,t)$ where $r = \sqrt{x^2+y^2}$ for a given point in space (x, y) in a $2D$ domain. Then, the cylindrically symmetric system will be given by:

$$\frac{\partial u}{\partial t} = b_f \phi_u^r \frac{u}{u + \frac{\mu_{fw}^r}{\mu_{fu}^r} w}(1-u-w)u - \mu_{fu}^r u + D\left(\frac{\partial^2 u}{\partial r^2} + \frac{1}{r}\frac{\partial u}{\partial r}\right), \quad \text{(A.1a)}$$

$$\frac{\partial w}{\partial t} = b_f \phi_w^r (1-u-w)w - \mu_{fw}^r w + D\left(\frac{\partial^2 w}{\partial r^2} + \frac{1}{r}\frac{\partial w}{\partial r}\right). \quad \text{(A.1b)}$$

To ensure a smooth transition across the singularity at $r = 0$, we impose Neumann boundary conditions, i.e.,

$$\frac{\partial u}{\partial r}\Big|_{r=0} = 0, \quad \frac{\partial u}{\partial r}\Big|_{r=L} = 0, \quad \text{(A.2a)}$$

$$\frac{\partial w}{\partial r}\Big|_{r=0} = 0, \quad \frac{\partial w}{\partial r}\Big|_{r=L} = 0, \quad \text{(A.2b)}$$

where L represents the radius of our circular domain. Note that this transformation of coordinates simplifies the 2-dimensional system in space into a 1-dimensional system for simulating a circular-shaped release protocol.

B Numerical Approximation of the 2-PDE System

The solution of the system of partial differential equations in A.1a–A.1b is approximated using a centered second-order finite difference method. Given a circular domain of radius L, we will denote $u_j^n \approx u(r_j, t_n)$ and $w_j^n \approx w(r_j, t_n)$ as the approximate solutions of the state variables at a radial grid point $r_j \in [0, L]$ and time $t_n \in [0, T]$, where T denotes the final time of the simulation. Assuming N is the number of subintervals at which we divide the domain $[0, L]$, the spatial grid point is defined as $r_j = (j - 1/2)h$, where $h = L/N$, for $j = 1, \ldots, N$. Thus, the system can be discretized as follows:

$$u_j^{n+1} \approx u_j^n + \Delta t\Big(b_f \phi_u^r \left(\frac{u_j^n}{u_j^n + (\frac{\mu_{fw}^r}{\mu_{fu}^r}) w_j^n}\right)(1-u_j^n-w_j^n)u_j^n - \mu_{fu}^r u_j^n + D\Delta_r u_j^n\Big), \quad \text{(B.3a)}$$

$$w_j^{n+1} \approx w_j^n + \Delta t\Big(b_f \phi_w^r (1-u_j^n-w_j^n)w_j^n - \mu_{fw}^r w_j^n + D\Delta_r w_j^n\Big). \quad \text{(B.3b)}$$

Here the time derivative is discretized using a Forward Euler Method, while the spatial derivative, which is present in the radial Laplacian term, is computed using centered differences:

$$\Delta_r u_j^n \approx \frac{u_{j+1}^n - 2u_j^n + u_{j-1}^n}{h^2} + \frac{1}{r_j}\left(\frac{u_{j+1}^n - u_{j-1}^n}{2h}\right) + O(h^2), \quad \text{(B.4a)}$$

$$\Delta_r w_j^n \approx \frac{w_{j+1}^n - 2w_j^n + w_{j-1}^n}{h^2} + \frac{1}{r_j}\left(\frac{w_{j+1}^n - w_{j-1}^n}{2h}\right) + O(h^2). \quad \text{(B.4b)}$$

The Neumann boundary condition at $r = 0$ can be enforced by assuming symmetry of the functions u and w about the origin. More precisely, the equations in A.2a–A.2b can be discretized as:

$$\frac{u_1^n - u_0^n}{2h} = 0, \quad \frac{u_{N+1}^n - u_N^n}{2h} = 0, \tag{B.5a}$$

$$\frac{w_1^n - w_0^n}{2h} = 0, \quad \frac{w_{N+1}^n - w_N^n}{2h} = 0, \tag{B.5b}$$

which imply $u_0^n = u_1^n$, $w_0^n = w_1^n$, $u_{N+1}^n = u_N^n$, and $w_{N+1}^n = w_N^n$. In Figure A, we present a graphical description of the spatial discretization implemented in our solver. Note

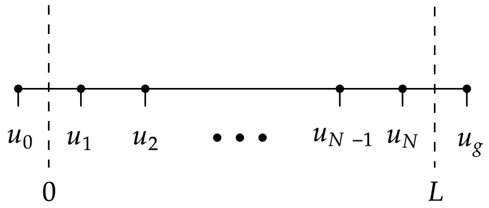

Fig A. Discretization diagram for computing the solution using centered differences, where u_g corresponds to a ghost point. We adjusted the grid points so that there is no grid point at $r = 0$, avoiding the singularity.

that with this discretization, the Laplacian at the boundary points $r = 0$ and $r = L$ will be computed based on the following cases. For $j = 1$:

$$\Delta u_1^n = \frac{u_2^n - 2u_1^n + u_0^n}{h^2} + \frac{u_2^n - u_0^n}{2hr_1} \tag{B.6a}$$

$$= \frac{u_2^n - u_1^n}{h^2} + \frac{u_2^n - u_1^n}{2hr_1}. \tag{B.6b}$$

Similarly, we can compute Δw_1^n. Now, for $j = N$, we have

$$\Delta u_N^n = \frac{u_N^n - 2u_N^n + u_{N-1}^n}{h^2} + \frac{u_N^n - u_{N-1}^n}{2hr_N}, \tag{B.7a}$$

$$\Delta w_N^n = \frac{w_N^n - 2w_N^n + w_{N-1}^n}{h^2} + \frac{w_N^n - w_{N-1}^n}{2hr_N}. \tag{B.7b}$$

Regarding the initial conditions of the system, we define them as follows:

$$u(r, 0) = c_0, \tag{B.8a}$$

$$w(r, 0) = ae^{-r^2/b^2}. \tag{B.8b}$$

Here, $u(r, 0)$ will be a constant function with the value c_0, which is determined by the carrying capacity of the mosquito population. Similarly, $w(r, 0)$ will be an inverse squared exponential function with real constants a and $b \neq 0$. The parameter a represents the height of the peak, while b controls the radius of the release shape. These two parameters also depend on the carrying capacity and determine the number of mosquitoes to be released in the field.

C Convergence Test of Numerical Approximation of Threshold of Infection

The root-finding algorithm presented in section 2.2 to characterize the threshold condition is sensitive to the time-step of the PDE solver. In Table A, we have computed

p_{thres} for three different time steps that differ consecutively by a factor of 1/2. We observe that the threshold condition among consecutive time steps reduces significantly and the infection curves will start to approach a single shape (see Figure B).

Table A. Convergence test of the numerical approximation of the threshold of infection Threshold values for three different PDE solver time steps. These values are expressed as the total number of infected mosquitoes released and their corresponding fraction of infection with respect to the female carrying capacity K_f. As the time step decreases by 50%, the difference between consecutive threshold values decreases.

Scaling Factor	Time Step	Total No. of Mosquitoes Released	Initial Fraction of Infection p_{thres}
Δt	0.017	1,072,640	0.357
$\Delta t/2$	0.008	1,047,040	0.349
$\Delta t/4$	0.004	1,032,140	0.344

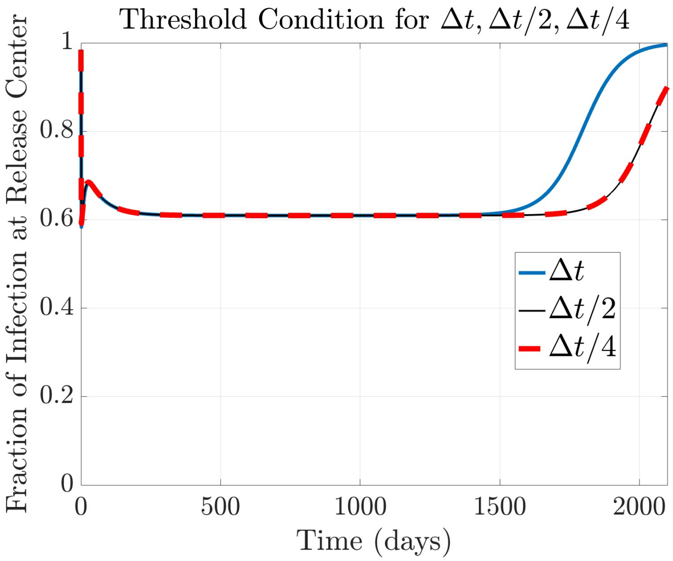

Fig B. Fraction of infection curves generated by releasing *Wolbachia*-infected mosquitoes at the threshold conditions determined by three PDE-solver time steps, Δt, $\Delta t/2$ and $\Delta t/4$, where the baseline time step value is $\Delta t = 1.78 \times 10^{-2}$. As the time-step decreases, the infection curves converge to a single shape whose initial condition corresponds to the numerical approximation of the threshold condition.

From these computations, it is possible to estimate the limiting value of the threshold condition as $\Delta t \to 0$. Let p^* denote this limiting value, then we can express the threshold conditions of Table A in terms of p^* as follows:

$$p_{thres}\Big(\Delta t\Big) = p^* + C\Big(\Delta t\Big)^m, \tag{C.9a}$$

$$p_{thres}\Big(\frac{\Delta t}{2}\Big) = p^* + C\Big(\frac{\Delta t}{2}\Big)^m, \tag{C.9b}$$

$$p_{thres}\Big(\frac{\Delta t}{4}\Big) = p^* + C\Big(\frac{\Delta t}{4}\Big)^m, \tag{C.9c}$$

for some power $m > 0$ and some constant $C > 0$. Now, if we compute the ratio

difference among consecutive threshold values, we get:

$$p_{thres}\Big(\Delta t\Big) - p_{thres}\Big(\frac{\Delta t}{2}\Big) = C(\Delta t)^m(2^m - 1)2^{-m}, \tag{C.10a}$$

$$p_{thres}\Big(\frac{\Delta t}{2}\Big) - p_{thres}\Big(\frac{\Delta t}{4}\Big) = C(\Delta t)^m 2^{-m}(2^m - 1)2^{-m}, \tag{C.10b}$$

$$\frac{p_{thres}\Big(\Delta t\Big) - p_{thres}\Big(\frac{\Delta t}{2}\Big)}{p_{thres}\Big(\frac{\Delta t}{2}\Big) - p_{thres}\Big(\frac{\Delta t}{4}\Big)} = \frac{1}{2^{-m}} = 2^m. \tag{C.10c}$$

From the previous equations, we can deduce the values for m and C:

$$m = \log_2\left(\frac{p_{thres}(\Delta t) - p_{thres}(\Delta t/2)}{p_{thres}(\Delta t/2) - p_{thres}(\Delta t/4)}\right) \approx 0.6781, \tag{C.11a}$$

$$C = \frac{p_{thres}(\Delta t) - p_{thres}(\Delta t/2)}{(\Delta t)^m(2^m - 1)2^{-m}} \approx 0.3277 \tag{C.11b}$$

Now, if we replace the values of m and C in C.9a, we obtain an estimation of p^*:

$$p^* = p_{thres}(\Delta t) - C(\Delta t)^m \approx 0.3357 \ \text{(fraction of infection)} \tag{C.12}$$

$$\approx 1,007,100 \ \text{(total number of mosquitoes)} \tag{C.13}$$
